# Smart Build-Plate for Metal Additive Manufacturing Processes

**DOI:** 10.3390/s20020360

**Published:** 2020-01-08

**Authors:** Adam Hehr, Mark Norfolk, Dan Kominsky, Andrew Boulanger, Matthew Davis, Paul Boulware

**Affiliations:** 1 Fabrisonic LLC, Columbus, OH 43221, USA; mnorfolk@fabrisonic.com; 2 Luna Innovations Inc., Blacksburg, VA 24060, USA; kominskyd@lunainc.com (D.K.); boulangera@lunainc.com (A.B.); davism@lunainc.com (M.D.); 3 Edison Welding Institute (EWI), Columbus, OH 43221, USA; pcboulware@gmail.com

**Keywords:** additive manufacturing, fiber optic, HD-FOS, UAM, L-PBF, embedded sensing

## Abstract

This paper discusses the development, processing steps, and evaluation of a smart build-plate or baseplate tool for metal additive manufacturing technologies. This tool uses an embedded high-definition fiber optic sensing fiber to measure strain states from temperature and residual stress within the build-plate for monitoring purposes. Monitoring entails quality tracking for consistency along with identifying defect formation and growth, i.e., delamination or crack events near the build-plate surface. An aluminum alloy 6061 build-plate was manufactured using ultrasonic additive manufacturing due to the process’ low formation temperature and capability of embedding fiber optic sensing fiber without damage. Laser-powder bed fusion (L-PBF) was then used to print problematic geometries onto the build-plate using AlSi10Mg for evaluation purposes. The tool identified heat generation, delamination onset, and delamination growth of the printed L-PBF parts.

## 1. Introduction

Additive manufacturing (AM) has rapidly evolved into a valuable technique for producing parts that, at times, cannot be fabricated through conventional machining methods. One challenge in AM is the lack of real-time feedback on the fabrication process and the quality of the part being made. This is especially critical given the relatively long periods of time that complex parts can require to be constructed. Hidden flaws in a part, which can be caused by excessive residual stresses, can result in the final part being unusable, and wasting valuable time, resources, and AM machine life.

At this point in time, damage events in parts are curtailed or quantified using experimental build-and-check standardization techniques. This standardization involves lengthy and costly testing, unique to a given application for a specific AM process. To minimize standardization time and cost, in-process control and monitoring approaches have recently begun to be developed using external techniques [1,2]. Light spectroscopy has been studied to link plume metrics to process stability, specifically through a line-to-continuum metric [3]. High-speed melt-pool imaging was evaluated to detect abnormal processing conditions through the application of machine vision, principal-component analysis, and k-means clustering [4]. Nondestructive evaluation techniques, including array eddy current and X-ray imaging, have been demonstrated for in situ monitoring of near-subsurface volumetric quality and process phenomena (e.g., melt-pool dynamics, solidification, keyholing), respectively [5,6]. High-resolution optical imaging of the build area with dynamic lighting scenarios was investigated to identify artificial lack-of-fusion defects [7]. Coaxial high-speed near-infrared imaging was investigated to visualize process dynamics and to measure high-temporal-resolution melt-pool-size metrics [8].

**Figure 1:**
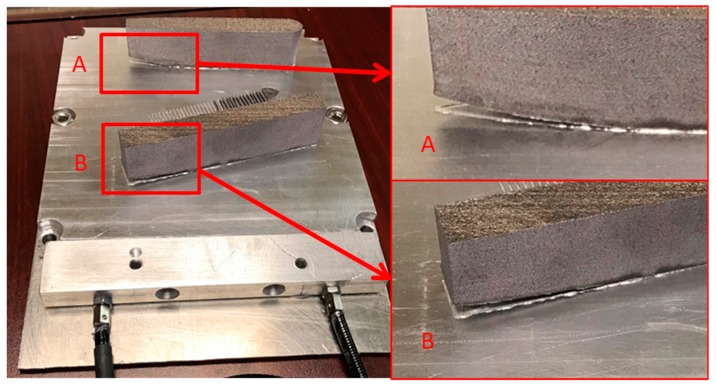
Delamination event from laser-powder bed fusion (L-PBF) evaluation in study. The components here were designed to intentionally delaminate. Delamination like this can occur on parts of interest, which often compromises the part.

These external AM monitoring approaches have shown promise to improve reliability, but the damage is often hidden from the user and cannot be detected with external sensing techniques, which limits their use. Instead, damage is not detected until obvious changes in the manufacturing process occur or when the part is removed, i.e., cracking and delamination caused from residual-stress accumulation. Experts identified that monitoring real-time internal information of metal AM parts is a critical need and gap in the technology [1,2]. Of utmost importance is the ability to identify internal residual stress, which leads to cracking and delamination. Often, this damage occurs between baseplate and build because of the high stress concentration in this location. Damage from residual stress accumulating between the baseplate and build is shown in Figure 1. Along with process-control and -monitoring technologies, advanced AM modeling tools are actively being developed to predict residual stress and other build challenges to minimize risk and lower cost. However, the integration of these models with on-board control remains limited and disjoint due to a lack in available sensing technologies for AM control.

To address this technology gap of lacking internal subsurface information, a build-plate with built-in sensors near the deposition surface can be developed. Strain is a useful parameter to measure since it combines information regarding temperature and stress within the build-plate, which may impart information about build quality that is not otherwise clear to the user. The approach taken by the team here involved building a continuous fiber optic strain sensor into a build-plate using ultrasonic additive manufacturing (UAM).

High-definition fiber optic sensors (HD-FOS) were used in this study. These sensors are made of glass, small in form factor, immune to electromagnetic radiation, interrogated remotely, and allow continuous monitoring along the fiber length. Fiber optic based strain sensors that were limited to external mounting on metallic structures can now be incorporated into structures with the advent of metal additive manufacturing [9,10,11,12]. UAM, a low-temperature metal additive-manufacturing technology, overcomes formation challenges found in directed energy deposition type processes and has successfully been used to build fiber optic Bragg gratings (FBGs) and HD-FOS into metal components for monitoring purposes [13,14,15,16,17,18].

**Figure 2:**
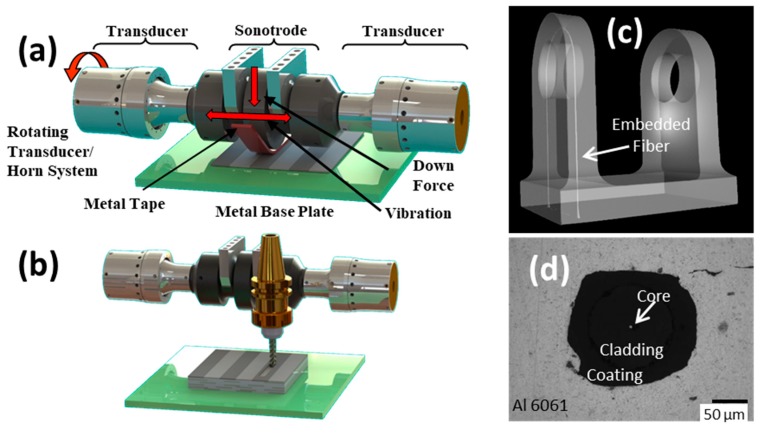
Embedding fiber optic strain sensors into metals using ultrasonic additive manufacturing (UAM): (**a**) Additive ultrasonic welding stage; (**b**) Subtractive computer numerical control (CNC) machining stage; (**c**) CT scan of aluminum bracket with embedded fiber around stress concentrator for damage monitoring, courtesy J. Sietins of Army Research Lab; (**d**) micrograph showing sensor consolidation into metal via UAM.

UAM uses high-frequency (ultrasonic) vibrations to scrub metal foils together in a synchronous manner. Computer numerical control (CNC) machining operations are periodically used with the ultrasonic-deposition stage to create internal features and finalize geometries [19]. Figure 2a details the ultrasonic-deposition stage, and Figure 2b details the CNC stage. Similar to ultrasonic metal welding, melting of the metal does not occur in the process [20]. Instead, the scrubbing action disrupts surface oxides and promotes metal lattice contact. The scrubbing action does generate heat on a localized level near the interface—measured to be near 150 °C for aluminum and copper alloys with fast-response embedded thermocouples slightly below the bond interface [21]. UAM enables the direct integration of temperature-sensitive components into the metal structure without damage, including fiber optic strain sensors. Figure 2c is a computed tomography (CT) scan of a bracket built with an integrated fiber optic sensor around the high-stress pin region. Figure 2d is a metallurgical cross-section of an embedded optical fiber. A portion of the interface is unconsolidated in the photo, which appears like a crack.

The aim of this paper was to discuss the design, manufacture, and evaluation of a “smart” build-plate or baseplate for monitoring internal residual stresses and temperatures near the build-plate/build interface. The paper begins by explaining how the device is built using UAM and HD-FOS. Data from the HD-FOS were interpreted and spatially mapped to the embedded fiber route using a custom MATLAB GUI. An open architecture laser-powder bed fusion (L-PBF) system was then used to print onto the smart baseplate for evaluation purposes and to compare with external monitoring techniques.

## 2. Materials and Methods

### 2.1. Build-Plate Manufacture

Low bend-loss polyimide coated HD-FOS fiber was used here. Polyimide coated fibers were chosen for this study due to commercial availability in the telecom industry and prior success in pilot studies. Strain measurements in the HD-FOS fiber were made using Rayleigh scatter from the intrinsic manufacturing defects during the fiber-draw process. The fiber was interrogated using optical frequency domain reflectometry (OFDR), an interferometric technique that can distinguish scattering points at different locations along the fiber [22].

**Figure 3:**
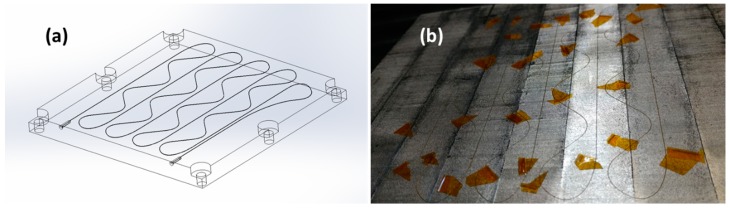
Fiber routing design of smart build-plate: (**a**) design showing internal fiber path; (**b**) fiber placement by inserting fiber into cut slot and using Kapton tape for temporary constraint during placement. Kapton tape is removed prior to consolidating. The internal fiber route used a strain Rosette pattern to measure three 2D strain states across the surface. Fiber was embedded close to the surface of printing to enhance sensitivity to surface strain events, i.e., delamination or cracking of the L-PBF samples.

To measure the strain behavior near the surface of the smart build-plate, the team designed a fiber route inspired by a rectangular strain rosette [23]. This design allowed measurement of the three 2D strain states to resolve magnitudes and directions. Spatial precision is a function of the furthest fiber spacing, which was 4 cm here. The fiber route is shown in Figure 3a. The fiber was embedded near the surface of the baseplate (0.64 mm below) to measure events like delamination and cracking between baseplate and printed parts.

The steps used to fabricate a smart build-plate were as follows:

1. Mount flat plate of aluminum alloy (AA) 6061-T6 on UAM vacuum chuck anvil.
2. Print layer of AA 6061-H18 (AA 6061-H18 is produced by following AA 3003 H18 rolling and annealing standard) foil material using UAM additive stage.
3. Cut in features for fiber embedding, including relief pockets and the fiber route using CNC stage.
4. Hand placement of the HD-FOS sensing fiber.
5. Vacuum AA 6061-H18 sheet over the fibers and consolidate in-place using the additive stage.
6. Inspect the embedded fiber using red light. If not broken, continue AA 6061-H18 material deposition to final height (~6 layers).
7. Final dimensioning of part using CNC stage.
8. Removal of part, deburr, and inspection of fiber using red light.

Steps 2 and 5 used printing parameters for AA 6061-H18 found to be optimal for strength and fiber consolidation from internal pilot studies. A preheat of 65 °C was used during printing. UAM processing parameters were 2 m/min for travel speed, 4000 N for applied down force, and 28 µm for side-to-side scrubbing of the ultrasonic tool.

The hand placement and consolidation of the fiber (Step 4) are shown in Figure 3b. The fiber was inserted into the cut channel and provided accurate location placement. The cut channel also prevented damage to the sensor during consolidation. Kapton tape was used intermittently to prevent the fiber from moving during placement. The tape was removed prior to overprinting and did not leave residue when ready to proceed to consolidation (Step 5).

Step 5 used a continuous piece of aluminum foil or sheet. This sheet was placed over the recently placed fiber and pulled down using an applied vacuum. A vacuum was used to ensure that the fiber remained in the channel during UAM. The vacuum was created by using a temporary gasket/putty material used in the composite bagging industry, a vacuum hose, and vacuum pump. A vacuumed sheet is commonly employed in the UAM process in addition to slit foil or tape.

**Figure 4:**
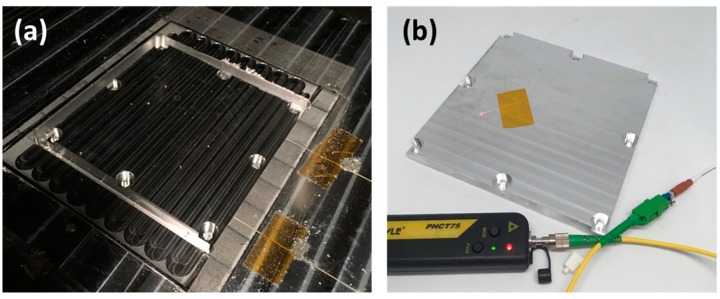
Final part dimensioning and inspection: (**a**) finishing operations on smart baseplate; (**b**) inspection of finished baseplate using red light. Strategic CNC stage cutting operation permitted embedding of fiber without damage and enabled part release from initial stock.

After the sensing fiber was integrated into the smart baseplate, final dimensioning was carried out using the CNC subtractive stage of the UAM process. Figure 4 shows the part prior and post removal from the UAM anvil. Red light was used to inspect the sensing fiber after removal to ensure a quality part. For handling and protection purposes, Hagitec^®^ tubing was placed around the embedded fiber. This tubing was mounted onto an intermediary bulkhead. This bulkhead was attached to the baseplate.

### 2.2. L-PBF and Data Sampling

Evaluation of the smart build-plate used an open-architecture laser-powder bed fusion (L-PBF) located at EWI. This L-PBF system was designed and fabricated to act as a platform for developing and evaluating both novel processing technologies, and novel monitoring and control methodologies for L-PBF. The system design was based on standard L-PBF functionalities present in commercial systems. However, this system allows modifications to be made to process parameters and enables measurement of the process. General process parameters, including laser power and scan speed, were configured in addition to controller-level parameters, including galvanometer accelerations and turn-around times. Part slicing and path planning were automated, but open to the user via numerous standard and nonstandard scan strategies. The system also provided open access to the optical delivery, build chamber, and baseplate assembly for melt pool, build plane, and baseplate sensing.

**Figure 5:**
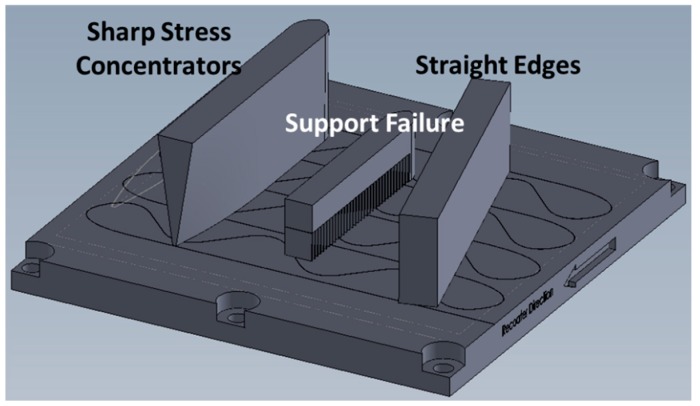
Planned L-PBF parts onto sensing fiber pattern. Builds entailed problematic features and were expected to fail by delamination or cracking due to high stress-concentrator features.

Design of the L-PBF parts for smart-build-plate evaluation, shown in Figure 5, was manipulated to induce part delamination, a problematic defect mode for L-PBF. Three iterations of standard block geometry were designed to introduce variable residual-stress scenarios at the baseplate-to-part interface. Varying aspect ratio was implemented to induce sharp stress concentrators. Varying support structure was implemented to induce delamination through poor part adherence to the baseplate. Standard block geometry, with no supports and straight edges, was included as a baseline.

The L-PBF parts were printed using AlSi10Mg powder with a Gaussian powder-size range of 15–45 µm. The powder was consolidated using laser power of 370 W, laser travel speed of 130 mm/s, 0.13 mm laser-track spacing, and powder thickness of 0.04 mm, laid by the recoater. To further promote delamination likelihood, the build-plate was not preheated in order to create a larger temperature differential in the build-plate, leading to enhanced internal stress.

**Figure 6:**
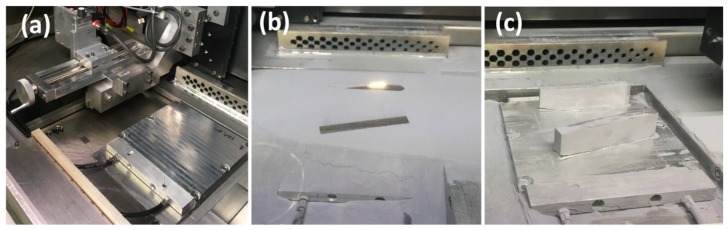
L-PBF printing: (**a**) smart build-plate loaded into open-architecture printer; (**b**) printing of features—parts were buried in spread powder, which limited external inspection; (**c**) removed powder showing delamination events.

The smart build-plate loaded into the open-architecture L-PBF printer ready for evaluation is shown in Figure 6a. Material deposition is shown in Figure 6b. The center support feature part shown in Figure 5 was abandoned at layer 63 (2.52 mm height) due to unwanted interference with the recoater. As powder was laid by the recoater, the interface between L-PBF parts and build-plate became buried in powder. Printing was continued until layer 632 (25.3 mm height). The build was prematurely aborted to inspect for potential defects once a potential qualitative delamination signature began to arise. Printed parts with the powder cleared away are shown in Figure 6c. Delamination and cracking can be seen between parts and the build-plate.

Concurrent with L-PBF part consolidation, HD-FOS measurements were taken during the printing process. Measurements were taken using an ODiSI 6000 series single-channel interrogator. This device used optical frequency domain reflectometry (OFDR) to make extremely sensitive measurements of the strain, aligned with the local axis of the fiber, at points spaced every 0.65 mm along the length of the fiber [22]. OFDR data were acquired with a wavelength scan range of 40 nm, and sample frequency was 20 Hz. Data were collected continuously throughout the L-PBF process to capture any transient failure events. Mechanical- and temperature-strain effects were measured simultaneously during this experiment.

## 3. Results and Discussion

**Figure 7:**
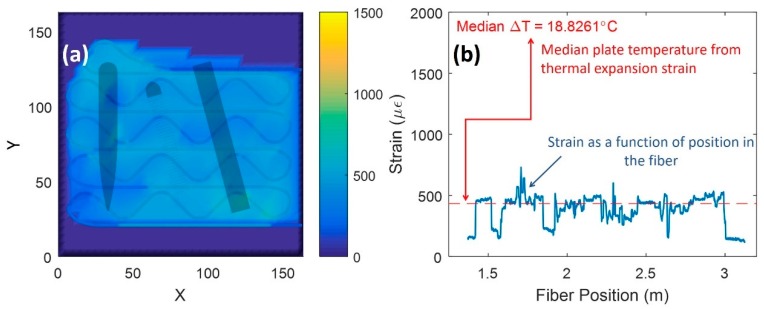
Spatial mapping of fiber data: (**a**) spatial map of fiber data with superimposed parts; (**b**) raw fiber data showing strain accumulation from heat generation from laser at layer 58.

Fiber optic data collected from L-PBF part printing were post-processed using MATLAB and CAD model data. Spatial mapping of the data at layer 58 to the internal fiber route is shown in Figure 7a while raw data are shown in Figure 7b. The median strain is attributed to build-plate temperature rise from printing. Some locations on the fiber were not as well-coupled to the build-plate, i.e., hair-pin turns, which creates a lower strain response with heat. The strain response shown is from the L-PBF process since the fiber strain was zeroed prior to starting.

**Figure 8:**
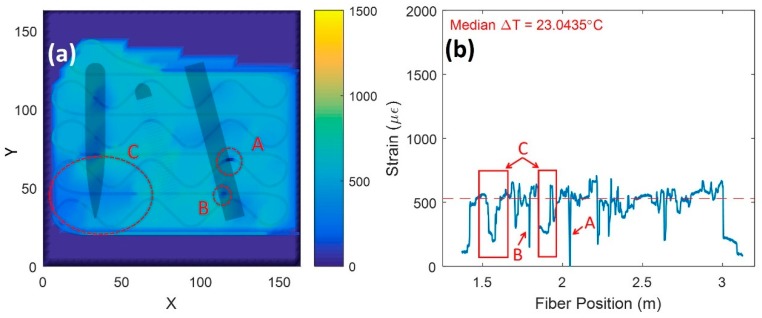
Damage-event data at layer 21: (**a**) spatial map showing strain response with localized areas of compression due to crack initiation; (**b**) raw fiber data showing strain accumulation from laser-heat generation.

**Figure 9:**
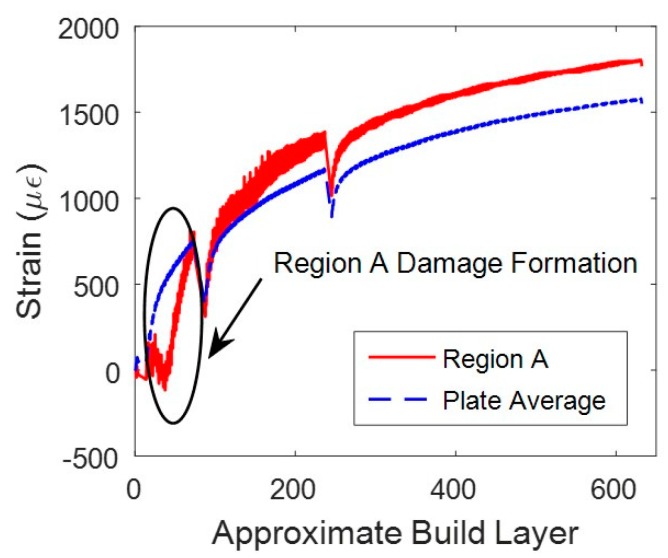
Region A strain compared to average plate strain. While damage was forming in region A, strain trend and magnitude were different than plate average. Once damage passed through region A, strain trend and magnitude became more like plate average. Dips in magnitudes were related to short breaks in printing process.

Evidence of build delamination is shown in Figure 8. These data were collected near layer 21. The data support the initiation and growth of a delamination or crack due to the measured net compressive-stress state in locations A, B, and C. This compressive state is expected from the shear-strain component and some portions of the normal strain components [24]. Figure 9 compares the strain at location A against the average plate strain during the L-PBF build. As crack or delamination grew in size near the fibers, net compressive strain state increased. On the other hand, the plate average continued to increase in tensile strain due to thermal expansion from the laser heating the build surface. Once the damage passed through region A, the strain returned to a tensile strain state similar to the plate average. The compressive states measured during the L-PBF build directly related to areas of delamination observed after the build was aborted (see Figure 1).

**Figure 10:**
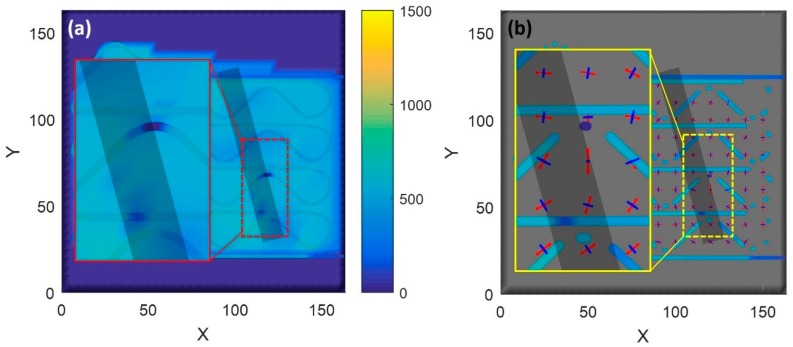
Analysis of strain magnitude and direction at layer 21: (**a**) high compressive strain near build edges believed to be a defect; (**b**) analysis of strain-vector field in same region as (**a**). Strain-vector direction changes also occurred near build defect.

Figure 10 evaluates the compression states of locations A and B from Figure 8 in more detail. Figure 10b is the full field strain map produced by the distributed rosette data in the smart build-plate. In order to produce this representation, raw data were partitioned into components that were aligned with the three key axes of the fiber (horizontal, +45°, and –45°). Each of those three sets of data was then interpolated with neighboring fibers and locations to establish averaged strain components at various locations within the sensing grid pattern. The data from the three components were then combined, just as it would be for a foil strain rosette, in order to determine the magnitude and orientation of the principal strain components. Principal tension is shown by the red outward-pointing arrows, and principal compression is shown by the blue inward-pointing arrows. For visualization purposes, the arrows were scaled to avoid overlap, so this representation depicts the relative strain fields at each point in the plate over time, but does not visually report the absolute magnitude of the stress components.

The strain field in Figure 10 was generally composed of equal tension (red arrow) and compression (blue arrow) areas due to even thermal-expansion effects. A few regions of particular note are visible in the data in Figure 10b that corresponded to the areas of compression or believed damage in Figure 10a. These areas are highlighted in the inset in Figure 10b. These highlighted areas showed the directional alignment of the strain vector with the damage state. This directionality was likely linked with delamination and crack-tip direction since the material was yielding and failing in those locations, i.e., location of principal strains. Additional work is required to fully understand the relationship between vector direction and damage-progression direction.

## 4. Summary and Technology Outlook

UAM was used to embed high-definition fiberoptic strain sensors (HD-FOS) into an AA 6061 build-plate for the laser-powder bed fusion (L-PBF) process. The motivation for this smart build-plate originated in the quality monitoring of L-PBF builds for defects such as delamination and cracking near the build-plate surface. An open architecture L-PBF system was used to evaluate the smart baseplate’s utility by printing problematic components using AlSi10Mg powder. The embedded HD-FOS provided information on delamination initiation and growth during the L-PBF print at the lower layers, while external-based indicators showed no defects. The team is continuing to improve upon this concept through enhanced fiber-routing designs and alternative alloy systems, e.g., stainless steels.
